# Dangerous Medicine: The Story Behind Human Experiments with Hepatitis

**DOI:** 10.3201/eid2907.230064

**Published:** 2023-07

**Authors:** Chari Cohen

**Affiliations:** Hepatitis B Foundation, Doylestown, Pennsylvania, USA

**Keywords:** hepatitis, clinical research, bioethics, viruses, hepatitis A, hepatitis B, hepatitis C, informed consent

Does the good of the public ever outweigh individual risk? What responsibility do scientists, research sponsors and the government have for clinical study participants, particularly those from vulnerable populations?

Dr. Sydney Halpern tackles these fundamental questions in *Dangerous Medicine: The Story Behind Human Experiments with Hepatitis*, as she tells the compelling and largely hidden story of the hepatitis experiments that took place in the United States during World War II and most of the Cold War (Figure). Those experiments involved infecting persons, primarily prisoners, conscientious objectors, and institutionalized, developmentally disabled children, with hepatitis. Throughout most of the experiments, scientists had not yet identified the distinct hepatitis viruses. Thus, while they justified their studies as the pursuit of ending hepatitis epidemics through development of a vaccine, they didn’t realize that they were infecting persons with a mix of hepatitis A, B, and C viruses. They also didn’t fully understand the ramifications; both hepatitis B and C could lead to chronic, lifelong infections that would increase a person’s lifetime liver cancer risk by as much as 40%.

**Figure:**
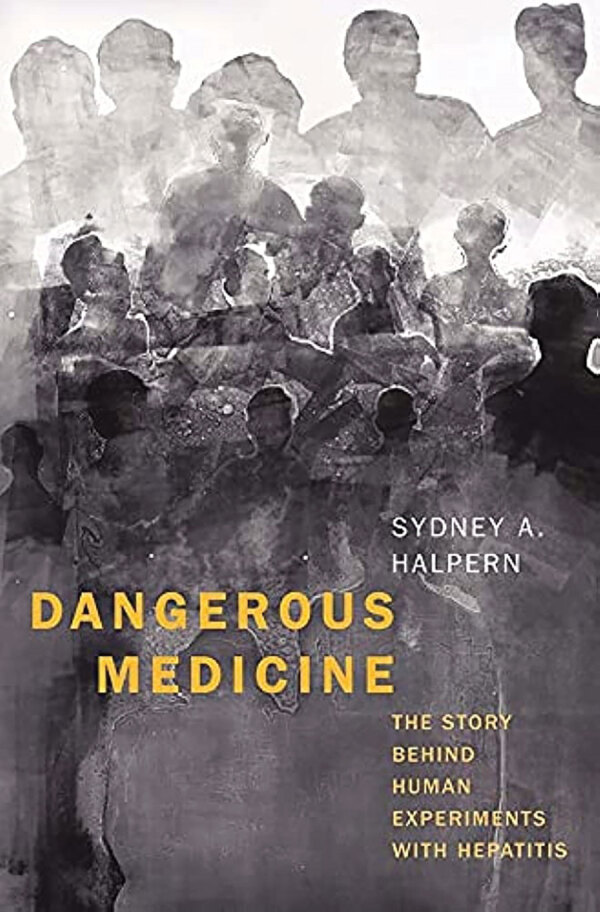
Dangerous Medicine: The Story Behind Human Experiments with Hepatitis

The author does an excellent job of setting up the sociopolitical climate that fostered the hepatitis experiments and of highlighting the roles that politics, media, the scientific elite, and wartime sentiment played. Science in the name of national defense created a moral imperative for persons who were not doing their duty as Americans (i.e., war objectors and prisoners) to contribute to society by volunteering their health. The recruitment of institutionalized children had a different, but still highly troubling, narrative, one of coercion and misinformation. Dr. Halpern expertly guides readers in an exploration of who controls the narrative, and thus the power.

The hepatitis studies played an important role in the rise of bioethics and creation of federal oversight of clinical research. Dr. Halpern discusses the complexities of informed consent and coercion and the ethics of research among vulnerable populations. The reader is asked to consider the responsibility (or lack thereof) that researchers and research sponsors hold to mitigate the long-term impacts of participating in clinical studies. She points out that we have not yet adequately explored the extent of the harms of the hepatitis experiments. We must continue to wrestle with the fundamental ethical questions inherent in clinical research to prevent creating such harmful situations now and into the future.

This book is important to read at any time, but perhaps no more so than today. As we face new infectious challenges like SARS-CoV-2, we run the risk of conducting clinical research in the dark, without knowing the actual risks and harms of studies to induce infection. A for-the-greater-good mentality is a slippery slope, which erroneously justifies deliberate risk in the face of public health crisis.

Dr. Halpern provides comprehensive detail throughout the book without losing sight of the narrative, and she weaves in perspectives from study participants themselves. In doing so, she crafts a well-balanced, authoritative, engaging story that will be appreciated by researchers, clinicians, public health professionals, and anyone interested in the history of science and bioethics.

